# Supplementary material for: A power law describes the magnitude of adaptation in neural populations of primary visual cortex
Source: Nat Commun. 2023 Dec 15;14:8366. doi: 10.1038/s41467-023-43572-w (PMC10724159; doi:10.1038/s41467-023-43572-w)
Supplement: Supplementary file 1 — Supplementary Information [file 41467_2023_43572_MOESM1_ESM.pdf]

Supplementary Information

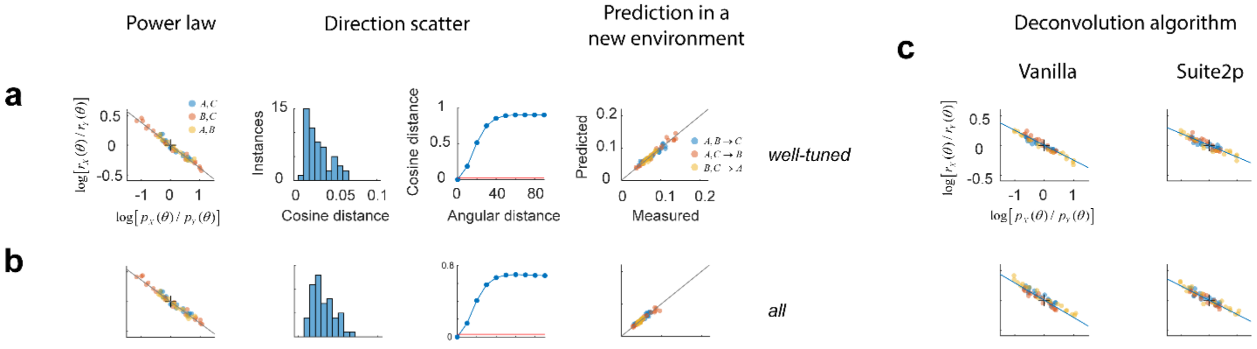

Supplementary Figure 1. Data selection and spike deconvolution algorithm hve little impact on the results. **a,** Analysis of a dataset by selecting well-tuned neurons with circular variance less than 0.5, and **b,** Analysis of the same dataset using all neurons. Only minor differences are observed. The cosine distance between response vectors corresponding to orientations 90 deg apart is lower when we include all neurons. The equivalent angular difference increases as well. Both the power law and its prediction remain largely unchanged. **c,** The power law is robust to the selection of deconvolution algorithms. Here we compared “Vanilla” and “Suite2p” as described in Berens, P., *et al* (2018). Community-based benchmarking improves spike rate inference from two-photon calcium imaging data. *PLoS computational biology*, *14*(5), e1006157.

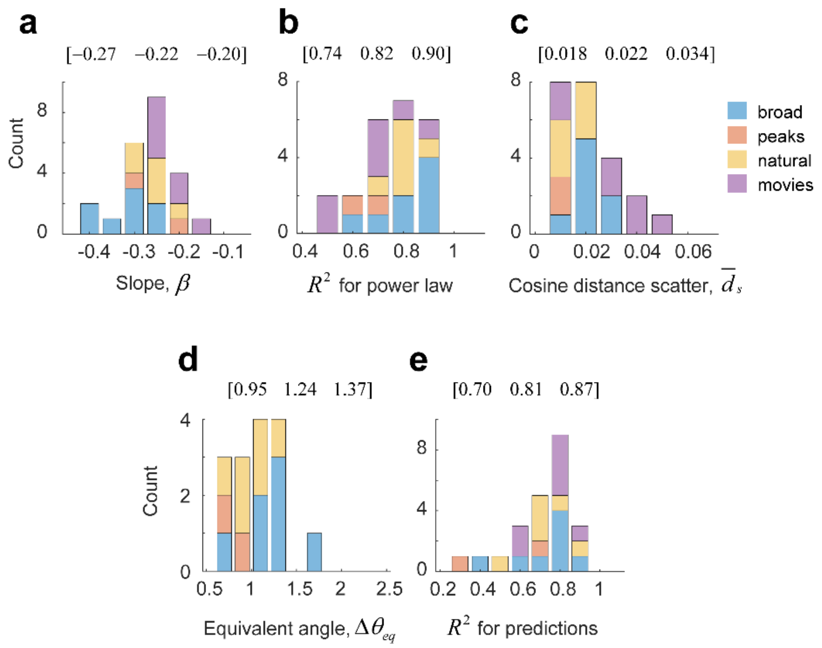

Supplementary Figure 2. Distribution of data fits**. a,** Distribution of the slope in the power law across all our experiments. Different experiment types are assigned different colors. Slopes were the largest for experiments using broad von Mises distributions and lowest using natural movie sequences. The numbers at the top indicate the 25th, 50th and 75th percentiles of the distribution. **b,** Distribution of the R-squared goodness of fit for the power law. **c,** Distribution of cosine distance scatter, **d,** Distribution of the equivalent angle, **e,** Distribution of R^2 values for the predictions in new environments. Note these values have almost the same distribution as in panel **b**.

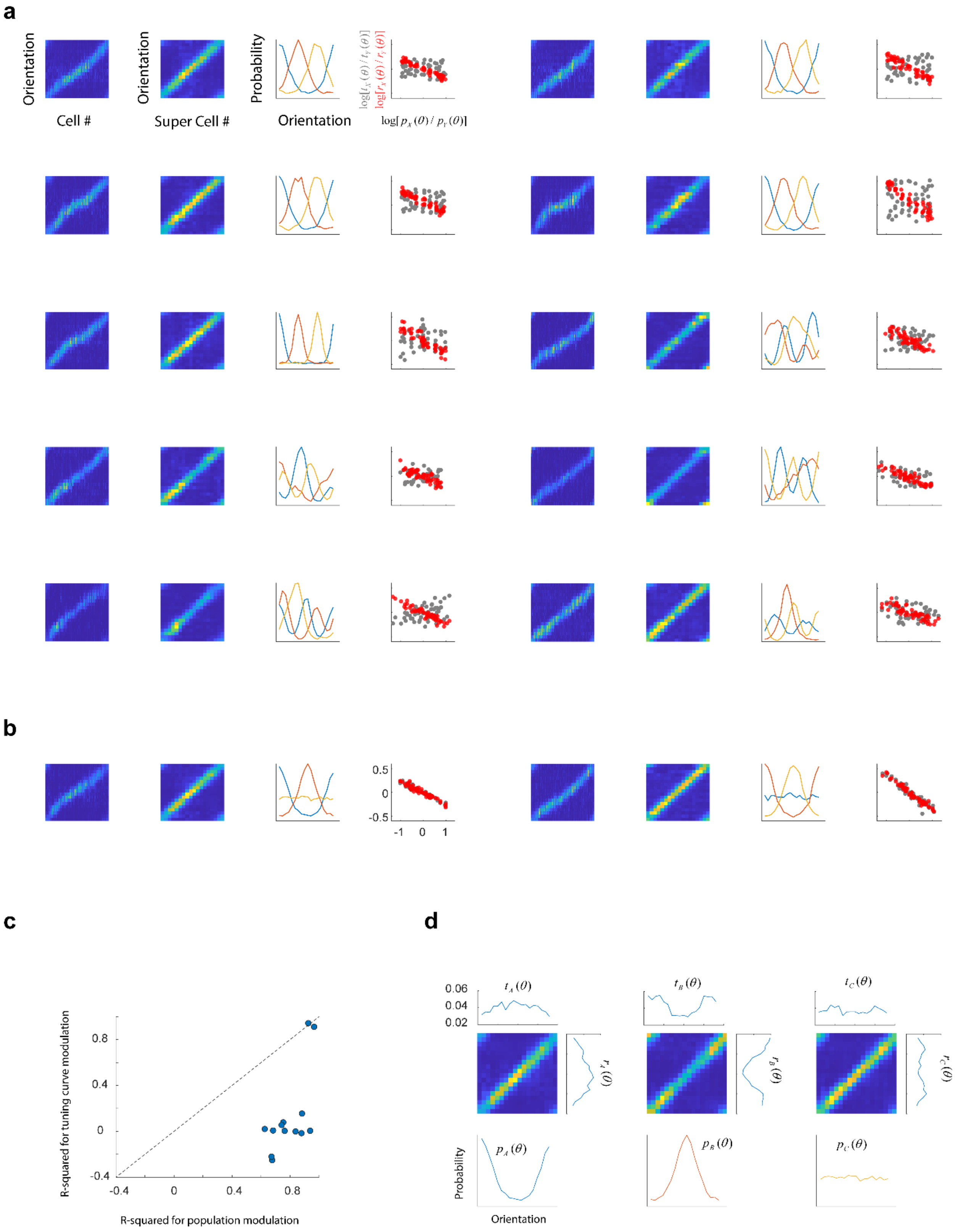

a
Orientation
Cell #
Orientation
Super Cell #
Probability
Orientation
$\log[t_X(\theta) / t_Y(\theta)]$
$\log[r_X(\theta) / r_Y(\theta)]$
$\log[p_X(\theta) / p_Y(\theta)]$
b
0.5
0
-0.5
-1
0
1
c
R-squared for tuning curve modulation
0.8
0.4
0
-0.4
-0.4
0
0.4
0.8
R-squared for population modulation
d
0.06
0.04
0.02
$t_A(\theta)$
$t_B(\theta)$
$t_C(\theta)$
$r_A(\theta)$
$r_B(\theta)$
$r_C(\theta)$
Probability
$p_A(\theta)$
$p_B(\theta)$
$p_C(\theta)$
Orientation

Supplementary Figure 3. Assessing the contributions of modulations in population versus modulations in tuning curves. These are analogous to the gain factors in a prior study[31]. **a**, Each set of four panels illustrates the analysis for a separate experiment. First, for each environment, we convert the matrix representing the responses for each cell, shown in the leftmost panel, into one where neurons are grouped by their preferred orientation in 18 different bins and the results averaged. Thus, the second panel in the row is a matrix of size 18 x 18 (18 orientations and 18 "super neurons"). The l_2 norms of the rows in this matrix are the population norms $r_X(\theta)$, where θ is the orientation of a stimulus. Similarly, we can take the l_2 norms of the columns, which represents the tuning curve norms, which we denote by $t_X(\theta)$. Here, θ represents the preferred orientation of a super-neuron. Suppose we adapt the population to an orientation θ. Is the change in the population best described by a change in $t_X(\theta)$ or a change in $r_X(\theta)$? More generally, do the ratios of the population magnitudes or the ratios of tuning curve magnitudes best correlate with the ratios of stimulus priors? The scatter plots show that the population magnitudes do a much better job than the tuning curve magnitudes. **b,** The only instances where the tuning curve magnitudes provided as good a prediction those of the population were in situations where the environments were simple von Mises distributions 90 deg apart with a uniform distribution. **c,** Across our experiments, the R^2 values are substantially higher when the population magnitudes are used compared to those of tuning curve magnitudes (except for the two isolated cases mentioned in **b**). **d**, The reasons that both population and tuning curve magnitudes are approximately the same in the simple environments shown in **b**, are the following. First, the matrix is nearly diagonal for super-cells that are well tuned. Second, the environments cause a modulation of responses along the diagonal. Third, this means that the norms of columns and rows are approximately the same. The effect along the diagonal, therefore, is not well-defined. Different combinations of population and tuning curve modulations can explain it. These analyses suggest that complex environments are better suited at discriminating between modulation of the population and modulation of the tuning curves. In our data, the former explanation prevails.

animal	type	sex	ncells	selected	gOSI 25	gOSI 50	gOSI 75	equiv_angle	mean_dth	beta	R2_pl	R2_pr	R2_pl_o	R2_pr_o	kopt	norm_a	R2_norm
adpt08_001_000	1	F	913	443	0.282	0.494	0.737	0.897	0.016	-0.251	0.930	0.801	0.944	0.813	3.360	0.507	0.898
adpt08_002_000	1	F	906	263	0.196	0.342	0.567	1.185	0.021	-0.291	0.897	0.803	0.899	0.808	23.357	0.522	0.925
adpt11_001_000	1	M	767	205	0.189	0.328	0.543	1.379	0.026	-0.380	0.972	0.945	0.978	0.963	8.859	0.504	0.936
adpt08_011_000	1	F	1001	304	0.231	0.377	0.582	1.220	0.020	-0.220	0.948	0.889	0.949	0.895	18.330	0.543	0.909
adpt08_012_000	1	F	689	161	0.165	0.283	0.489	1.877	0.030	-0.259	0.885	0.754	0.890	0.773	11.288	0.545	0.895
adpt09_070_000	1	F	856	275	0.206	0.351	0.607	1.480	0.024	-0.240	0.772	0.601	0.775	0.608	18.330	0.528	0.928
adpt09_071_000	1	F	675	124	0.161	0.277	0.457	2.369	0.035	-0.365	0.693	0.488	0.693	0.488	100.000	0.544	0.935
adpt09_081_000	1	F	724	186	0.181	0.313	0.528	1.342	0.023	-0.307	0.865	0.689	0.902	0.817	4.281	0.516	0.933
adpt09_100_000	2	F	764	288	0.211	0.385	0.639	0.774	0.014	-0.169	0.249	-0.211	0.789	0.729	2.637	0.497	0.840
adpt09_101_000	2	F	650	150	0.160	0.292	0.493	1.020	0.018	-0.251	0.219	-0.694	0.676	0.381	4.281	0.484	0.868
adpt10_010_000	3	F	534	149	0.190	0.309	0.560	0.955	0.017	-0.255	0.785	0.547	0.831	0.713	4.281	0.498	0.764
adpt10_050_000	3	F	500	151	0.168	0.310	0.590	0.800	0.014	-0.203	0.716	0.639	0.818	0.772	2.637	0.512	0.944
adpt10_060_000	3	F	501	134	0.164	0.294	0.533	0.949	0.018	-0.200	0.753	0.526	0.821	0.568	3.360	0.486	0.760
adpt09_130_000	3	F	644	177	0.197	0.337	0.558	1.357	0.022	-0.258	0.922	0.920	0.928	0.932	18.330	0.463	0.844
adpt09_150_000	3	F	984	239	0.153	0.272	0.495	1.257	0.022	-0.224	0.701	0.503	0.830	0.707	3.360	0.487	0.883
adpt11_011_000	3	M	459	118	0.188	0.317	0.522	1.264	0.022	-0.187	0.738	0.820	0.754	0.722	1.000	0.522	0.962
adpt09_500_000	4	F	1287	1287					0.043	-0.204	0.808	0.876				0.615	0.931
adpt09_501_000	4	F	1286	1286					0.053	-0.198	0.582	0.634				0.676	0.875
adpt10_200_000	4	F	657	657					0.039	-0.206	0.705	0.857				0.679	0.935
adpt10_500_000	4	F	1379	1379					0.036	-0.210	0.731	0.699				0.622	0.900
adpt10_700_000	4	F	457	457					0.017	-0.218	0.903	0.900				0.429	0.829
adpt11_012_000	4	M	490	490					0.050	-0.133	0.599	0.826				0.701	0.647
adpt11_030_000	4	M	601	601					0.016	-0.178	0.770	0.937				0.496	0.871

Supplementary Table 1. Summary of experiments. Each row summarizes a different experiment. From left to right, the columns include the following information: experiment name, sex, type of experiment (codes: 1, von Mises and uniform distributions; 2, peaked distributions; 3, natural orientation distributions; 4, movie sequences), total number of cells recorded, total number of cells selected (with $\mathrm{gOSI} > 0.5$), 25th percentile of gOSI, 50th percentile of gOSI, 75th percentile of gOSI, equivalent angle, mean cosine distance scatter, β fit to the power law, R-squared value for the power law relationship, R-squared value for the prediction, R-squared value for the power law relationship after smoothing correction, R-squared value for the prediction after smoothing correction, optimal κ, constant of proportionality between l_1 and l_2 norms, R-squared value for the linear fit between the norms.
