# Supplementary material for: A power law describes the magnitude of adaptation in neural populations of primary visual cortex
Source: Nat Commun. 2023 Dec 15;14:8366. doi: 10.1038/s41467-023-43572-w (PMC10724159; doi:10.1038/s41467-023-43572-w)
Supplement: Supplementary file 3 — Reporting Summary [file 41467_2023_43572_MOESM3_ESM.pdf]

nature portfolio

Corresponding author(s): Dario L. Ringach

Last updated by author(s): Oct 10, 2023

Reporting Summary

Nature Portfolio wishes to improve the reproducibility of the work that we publish. This form provides structure for consistency and transparency in reporting. For further information on Nature Portfolio policies, see our Editorial Policies and the Editorial Policy Checklist.

Statistics

For all statistical analyses, confirm that the following items are present in the figure legend, table legend, main text, or Methods section.

n/a	Confirmed	
☐	☒	The exact sample size (*n*) for each experimental group/condition, given as a discrete number and unit of measurement
☐	☒	A statement on whether measurements were taken from distinct samples or whether the same sample was measured repeatedly
☐	☒	The statistical test(s) used AND whether they are one- or two-sided<br>*Only common tests should be described solely by name; describe more complex techniques in the Methods section.*
☐	☒	A description of all covariates tested
☐	☒	A description of any assumptions or corrections, such as tests of normality and adjustment for multiple comparisons
☐	☒	A full description of the statistical parameters including central tendency (e.g. means) or other basic estimates (e.g. regression coefficient) AND variation (e.g. standard deviation) or associated estimates of uncertainty (e.g. confidence intervals)
☐	☒	For null hypothesis testing, the test statistic (e.g. *F*, *t*, *r*) with confidence intervals, effect sizes, degrees of freedom and *P* value noted<br>*Give P values as exact values whenever suitable.*
☒	☐	For Bayesian analysis, information on the choice of priors and Markov chain Monte Carlo settings
☒	☐	For hierarchical and complex designs, identification of the appropriate level for tests and full reporting of outcomes
☒	☐	Estimates of effect sizes (e.g. Cohen's *d*, Pearson's *r*), indicating how they were calculated

Our web collection on statistics for biologists contains articles on many of the points above.

Software and code

Policy information about availability of computer code

Data collection	Matlab (R2023a), Scanbox (Tower v3)
Data analysis	Matlab (R2023a). Sample Matlab code describing the structure of the database and the replication of some of our analyses can be found along with the data at https://figshare.com/s/47275dd78f37230f7c83.

For manuscripts utilizing custom algorithms or software that are central to the research but not yet described in published literature, software must be made available to editors and reviewers. We strongly encourage code deposition in a community repository (e.g. GitHub). See the Nature Portfolio guidelines for submitting code & software for further information.

Data

Policy information about availability of data

All manuscripts must include a data availability statement. This statement should provide the following information, where applicable:

- Accession codes, unique identifiers, or web links for publicly available datasets
- A description of any restrictions on data availability
- For clinical datasets or third party data, please ensure that the statement adheres to our policy

Data including the mean responses of the population for each experiment can be found in the Figshare repository at https://figshare.com/s/47275dd78f37230f7c83.

Research involving human participants, their data, or biological material

Policy information about studies with human participants or human data. See also policy information about sex, gender (identity/presentation), and sexual orientation and race, ethnicity and racism.

Reporting on sex and gender	N/A
Reporting on race, ethnicity, or other socially relevant groupings	N/A
Population characteristics	N/A
Recruitment	N/A
Ethics oversight	N/A

Note that full information on the approval of the study protocol must also be provided in the manuscript.

Field-specific reporting

Please select the one below that is the best fit for your research. If you are not sure, read the appropriate sections before making your selection.

☒ Life sciences ☐ Behavioural & social sciences ☐ Ecological, evolutionary & environmental sciences

For a reference copy of the document with all sections, see nature.com/documents/nr-reporting-summary-flat.pdf

Life sciences study design

All studies must disclose on these points even when the disclosure is negative.

Sample size	Number of cells within an imaged volume was maximized by having the largest field of view possible that still provided good segmentation and signal to noise of individual neurons. The statistical significance of the power law can then be demonstrated within individual experimental sessions.
Data exclusions	Neurons that were not well tuned were excluded from our analyses. The exclusion criteria is provided in "Statistics and Reproducibility" under the methods. We note, however, that the Figshare repository includes all the data prior to selection.
Replication	Our findings are observable at the level of individual experimental sessions. A total of n=16 independent experiments showed a significant effect replicating the same phenomena (see Supplemental Table 1)
Randomization	N/A. The design does not involve allocation of subjects to different groups.
Blinding	N/A. There are no treatment groups. Therefore, no blinding.

Reporting for specific materials, systems and methods

We require information from authors about some types of materials, experimental systems and methods used in many studies. Here, indicate whether each material, system or method listed is relevant to your study. If you are not sure if a list item applies to your research, read the appropriate section before selecting a response.

Materials & experimental systems

n/a	Involved in the study	
☒	☐	Antibodies
☒	☐	Eukaryotic cell lines
☒	☐	Palaeontology and archaeology
☐	☒	Animals and other organisms
☒	☐	Clinical data
☒	☐	Dual use research of concern
☒	☐	Plants

Methods

n/a	Involved in the study	
☒	☐	ChIP-seq
☒	☐	Flow cytometry
☒	☐	MRI-based neuroimaging

Animals and other research organisms

Policy information about studies involving animals; ARRIVE guidelines recommended for reporting animal research, and Sex and Gender in Research

Laboratory animals	A total of 5 mice were used in these experiments.
Wild animals	No wild animals were used in these studies.
Reporting on sex	Four mice were male and one female.
Field-collected samples	No field collected samples/animals were used.
Ethics oversight	The procedures in the experiments described here were approved by UCLA's Office of Animal Research Oversight (the Institutional Animal Care and Use Committee) and were in accord with guidelines set by the U.S. National Institutes of Health.

Note that full information on the approval of the study protocol must also be provided in the manuscript.
